# Achieving climate sustainability in the Republic of Congo: The role of economic growth, biomass energy consumption, rule of law and government effectiveness- a NARDL Approach

**DOI:** 10.1016/j.heliyon.2024.e34256

**Published:** 2024-07-09

**Authors:** Railh Gugus Tresor Massonini Ngoma, Xiangqian Wang, Xiang Rui Meng, Cety Gessica Abraham Mahanga Tsoni, Sumaiya Bashiru Danwana, Benjamine Tsoni Ndombi

**Affiliations:** a School of Economics and Management, Anhui University of Science and Technology, No. 168 Taifeng Road, Huainan, 232001, China; b School of Humanities and Social Science, Anhui University of Science and Technology, No. 168 Taifeng Road, Huainan, 232001, China

**Keywords:** CO2 emissions, Biomass energy consumption, Non-linear ARDL, Republic of Congo

## Abstract

This study explores the relationship between economic growth (GDP), biomass energy consumption (BEC), Rule of Law, and Government Effectiveness on climate change (CO2 emissions) in the Republic of Congo from 1990 to 2020. We employed a nonlinear autoregressive distributed Lag (NARDL) model to analyse data from World Bank databases. Higher GDP leads to lower CO2 emissions in the long run. Increased BEC also reduces emissions, but a decrease can have a small negative impact. Interestingly, a stronger Rule of Law and Government Effectiveness is associated with higher CO2 emissions in the short run, potentially due to relaxed environmental regulations. However, a stronger Rule of Law and Government Effectiveness leads to lower emissions in the long run, suggesting a potential shift towards sustainable practices. These findings provide valuable insights for policymakers aiming to achieve economic growth and climate stability in the Republic of Congo.

## 1 Introduction

In response to pressing climate concerns, many governments are stepping up efforts to mitigate the dependence on the main sources of carbon dioxide emissions by switching from fossil fuels to renewable energy. Against this backdrop, the Republic of Congo has established a goal of decreasing greenhouse gas emissions and dealing with global climate change through improved energy management, in line with actions made at the United Nations Framework Convention on Climate Change, the Paris Agreement, the Kyoto Protocol, and other climate mitigation goals. Renewable energy, particularly biomass energy, is essential in decarbonization [[1], [2], [3], [4]]. Therefore, it is necessary to expand this market to all regions of the world, especially Congo, as it is of significant interest to the Republic of the Congo, especially in energy management, which is a critical factor in its economic growth and maintaining climate health. Countries have historically relied heavily on fossil fuels such as natural gas, oil, coal, and nuclear power for various purposes, resulting in significant climate change and environmental degradation. Countries' long-standing reliance on fossil fuels has had a significant environmental impact, hastening climate change and contributing to frequent environmental damage. It has brought up questions regarding the long-term viability of the present energy practices and the need to move to cleaner, alternative forms of energy [[5], [6], [7]]. Therefore, it can be seen that with economic growth and development, our era has experienced several climatic transformations and has deteriorated significantly, affecting ecosystems, biodiversity, and human health and also exacerbating global warming [8]. Just think of the Industrial Revolution, marked by the exploitation of natural resources for energy, the proliferation of automobile and air transport powered by widespread oil use, and an increasing reliance on electricity. These are all examples of this trend [9,10].

Today, however, energy development is not only associated with economic progress but also with the many threats to our environment or climate. In addition to these risks, there is now the danger of global warming caused by carbon dioxide emissions. Scientists believe that higher economic growth in a country with high energy consumption can increase the demand for energy to meet the population's and industry's growing needs. This increased demand for energy may result in excessive exploitation of natural resources, including carbon-rich ecosystems like forests. This unregulated exploitation can lead to deforestation and other ecosystem degradation, accelerating biodiversity loss and exacerbating climate change [[11], [12], [13], [14], [15]]. As a result, while economic growth can spur development, it poses significant environmental risks if not managed sustainably. Policies and economic practices incorporating natural resource preservation and carbon emission reduction are required to promote sustainable and balanced economic development. The 1973 oil crisis taught us that energy dependence depends on fossil fuel depletion [16]. Cheah Wai Yan [17] argues that we must massively switch to renewable energy and energy efficiency. The solution to controlling carbon emissions seems simple: replace fossil fuels with renewable energies. Unlike fossil fuels, renewable energies are a remarkable source of energy that can be produced from inexhaustible resources [[18], [19], [20], [21], [22]]. A minimal discharge of waste accompanies their operation. Healthy energy is essential to development, economic growth, and reducing greenhouse gas emissions [23]. Clean energy innovation is necessary and critical for addressing today's environmental and energy challenges. Progress in this direction has the potential to significantly reduce global dependence on fossil fuels and mitigate the adverse effects of greenhouse gas emissions. Take solar panels as an example; as the efficiency of solar energy technology improves, the energy market becomes more competitive. Despite these advancements, challenges persist, such as high initial costs and integration with existing infrastructure. Investment in R&D is thus critical to overcoming these barriers and making solar technology more accessible and cost-effective on a large scale. Additionally, wind turbines are increasingly crucial in transitioning to renewable energy sources. Turbine design advancements, such as size and efficiency optimisation, can increase energy production capacity while lowering manufacturing and installation costs. However, questions remain about the environmental impact, particularly on natural landscapes. To ensure that wind installations are both sustainable and ethical, a comprehensive approach is required, which includes thorough ecological assessments and community consultations. Transitioning to renewable energy also necessitates investments in smart infrastructure and networks. Managing the intermittent flow of energy generated by sources such as solar and wind necessitates sophisticated storage and distribution networks. Furthermore, integrating energy management technologies like smart meters and automated distribution networks can improve resource utilisation and reduce energy losses. Clean energy production and storage innovations can transform Congo's energy landscape significantly. However, realising this potential necessitates proactively addressing this transition's technical, environmental, and social challenges. A successful energy transition is a more sustainable and resilient future requiring ongoing research, development, and investments in economic growth.

**Fig. 1:**
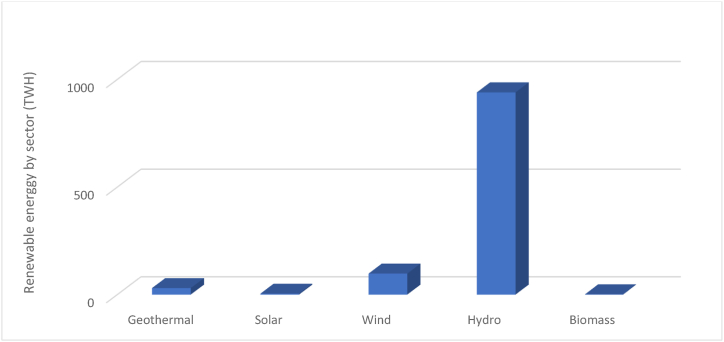
Energy by sector.

Source: Energy Information Administration, the African Development Bank

[24,25] But today, the performance and reliability of renewable energy are improving so much that they can now compete with previous energy sources (non-renewable energy sources) [26,27]. Thus, their distribution is made into five significant families according to their origin (solar, wind, hydraulic, geothermal, and biomass) (Fig. 1). The representation presented allows for an extensive evaluation of various energy systems based on how they are consumed, pointing out the crucial effect of multiple factors, such as natural resource availability and energy demands, on the energy mix. Notably, renewable energies have emerged as critical components of the Republic of Congo's energy landscape, highlighting the country's significant renewable resource potential as a catalyst for responsible and sustainable economic development. This article emphasises biomass energy among these renewable options due to its suitability, importance, and promise in combating climate change. Regardless of the Republic of Congo's current lack of biomass energy utilisation, improving this aspect has enormous potential for combating climate change while promoting economic growth and environmental sustainability. Biomass, made from organic byproducts such as agricultural residues, forestry waste, and organic municipal waste, provides economic opportunities and energy security benefits, facilitates effective waste management, promotes rural development, and improves technology transfer and capacity building. Finally, prioritising biomass energy represents a multifaceted strategy for advancing the Republic of Congo's sustainable development agenda, providing tangible benefits in the economic, environmental, and social domains while addressing pressing energy challenges and encouraging inclusive growth. When burned, the chemical energy of biomass is released as heat, which can be converted into biofuel and biogas and ultimately into useable energy sources such as fuel, electricity, or heat [28].

Using biomass has become essential for economic development and a better climate. Renewable energy consumption in developed industries increased from 27 % in 2019 to 29 % in 2020 [1.2.3.4]. Biomass, in its transformation, makes it possible to obtain more common fuels capable of maintaining our climate in good health [29]. With a vast original production of plant and animal waste, exploring biomass in all its forms has become essential. For example, biodiesel could be a promising alternative fuel to address the climate problem in the Republic of Congo. On the forest industry's side, bio-refining is the added value to significant changes in its activities to ensure its sustainability and development on international markets. In addition to having large quantities useable for this new industry route, considerable volumes are still underused. Therefore, the state must seize this opportunity since its production can be decentralised according to local energy resources [30]. As an organic molecule, it has multiple properties that are promising for many synthetic reactions, especially regarding environmental stability. Fossil fuels have been the primary source of energy for most economies. This is why research on this future new energy vector is imperative. The conversion of biomass into energy can represent sustainable development [31]. This energy can then be used as a fuel for heating, power generation, stationary applications, and transportation or as an additive to improve the energy efficiency of machinery [32].

**Fig. 2:**
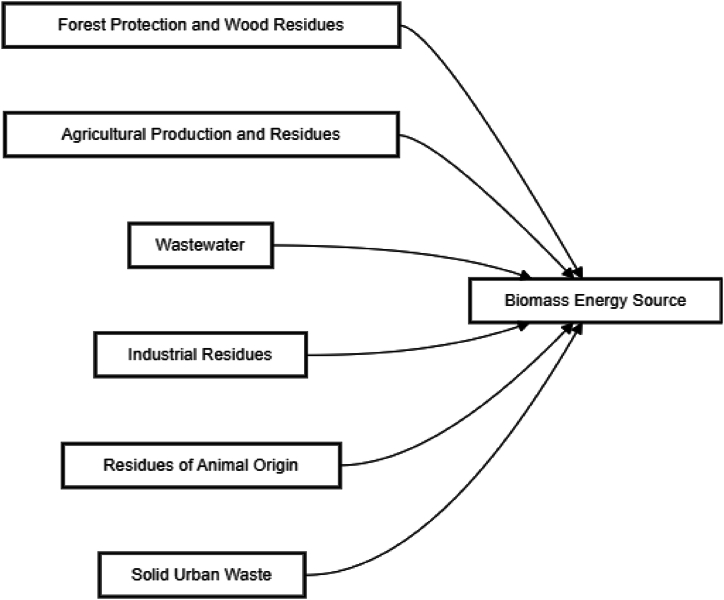
Biomass energy sources.

Source: OECD. Stat

The Republic of Congo has the resources and assets to develop biomass energy, as the country has abundant renewable biomass resources (Fig. 2) and the means to use them to create clean energy and materials. Its biomass residues come from the forestry, agricultural, and municipal waste industries. The quality of the residues available in the Republic of Congo is significantly higher than the remaining plant residues. In the past, collecting and processing this energy was impossible. But this is about to change since many developed countries have already started this type of flight [33,34]. By using these abundant and diverse organic residues in multiple regions, the Republic of Congo will be the first country in Africa to have a promising supply source for pure and healthy energy production. In addition, biomass energy can also be converted into solid, liquid, or gaseous energy carriers, giving rise to a wide range of bioenergy applications [35]. It can be burned directly, provide heat, electricity, biochemical, catalytic, or thermal conversion, and produce liquid fuels such as ethanol and renewable diesel. It can also produce gaseous fuels by digestion, gasification, and pyrolysis to obtain pyrolysis oils and high-value chemicals [[36], [37], [38]].

In the Republic of Congo, biomass could be divided into three categories: forestry, agribusiness, and urban. Forest biomass is the most widespread resource but still has good development potential in exploitation residues. Forest biomass–firewood, wood processing residues (bark, sawdust and planning, cuttings, offcuts, sludge from paper mill water treatment plants), and logging residues (branches, needles, leaves, stumps, crowns). However, to promote the development of biomass for energy production, it is essential to secure the material supply, which currently depends on timber harvesting, supply contract beneficiaries, and forest management. Regarding agri-food biomass, plant and animal residues from agricultural production and the agri-food processing industry should be encouraged. Regarding urban biomass, municipal wastewater and putrescible organic matter treatment plants in the residential, municipal, commercial, and institutional sectors must always be the subject of research efforts to improve their efficiency. To increase the social acceptance of CHP plants in urban areas, it is necessary to organise information and consultation sessions with the relevant groups [39]. There are different processes for energy recovery from biomass, depending on the resource type and the intended use [40].

**Fig. 3:**
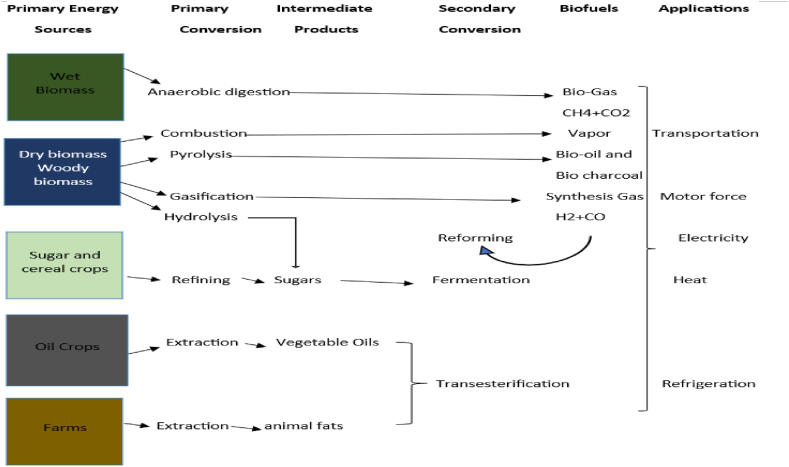
Biomass energy recovery.

Source: https://www.hydroquebec.com/data/developpement-durable/pdf/fiche-biomasse.pdf

In the Republic of Congo, the combustion of solid biomass is not widely used; bio-mechanization and gasification would be interesting to develop for stabilising organic matter by fermentation in the absence of oxygen, depending on the nature of the substrate used (see Fig. 3). It can be produced in a bioreactor or extracted from landfill cells. Biomass-derived gasification is manufacturing combustible gases by reacting solid or liquid fuels with a gasifying agent, such as air or oxygen, at atmospheric pressure. Biomass is transformed in whole or partially, mainly by heat, to produce a combustible gas. It consists mainly of hydrogen and carbon monoxide, with minimal amounts of methane, CO_2_, and tar. Gasification is an innovative alternative that consists, through a thermochemical process, of transforming solid biomass into combustible gas recoverable in multiple ways. This process transforms the raw material into a gas, which will be used as fuel. In the Republic of Congo, there are no biomass gasification plants. However, biomass power plants play a significant role in regulating CO_2_ emissions. Developing biomass energy, which undeniably has more advantages than disadvantages, would also be possible to reduce CO_2_ and grow the economy.

The thermochemical conversion process of biomass (pyrolysis) is the critical first step in all thermochemical reactors. It is carried out in an oxygen-free or hypoxic atmosphere to avoid oxidation and combustion [41]. Rapid pyrolysis products can be gaseous, liquid, or solid [42,43]. Gaseous products include H_2_, CH_4_, CO, CO_2_, and other gases, depending on the type of biomass. Liquid products include tars and oils that remain liquid at room temperature and acetone, acetic acid, etc. [36]. The solid product consists mainly of carbonised residues, pure carbon, and inert substances. Hydrogen can be produced directly by rapid pyrolysis as follows. If the temperature and residence time are sufficient:

Biomass + heat → H_2_ + CO_2_ + CH_4_ + other products

Methane and other hydrocarbons produced in the first reaction can be converted to H_2_ by a steam reforming process: CH_4_+HO_2_→CO+3H2.

**Fig. 4:**
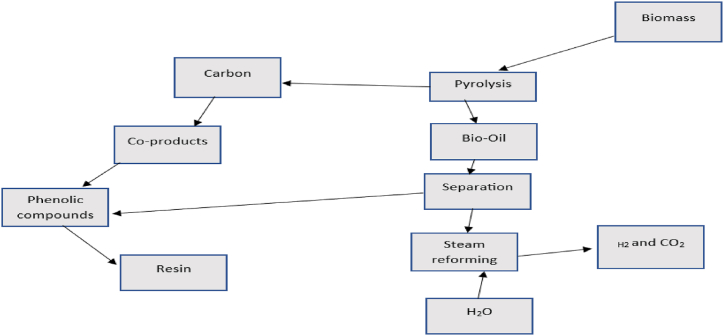
Thermochemical conversion of biomass.

Source: Author from author data [36,44].

A water-gas conversion reaction can be used to convert CO to CO_2_ and produce even more H_2_: CO + H _2_→CO_2_+H_2_ as shown in the following Fig. 4.

Biomass energy could contribute to the country's economic, social, and environmental development. Like what.

✓ The community benefits of waste reduction and associated environmental impacts.
✓ Proceed under technical regulations for the biomass processing industry and combined heat and power plants from biomass.
✓ Access to energy for the entire population under sustainable conditions and at affordable prices.
✓ The waste is produced by each living species, so their availability is unlimited [45].

In addition to the energy mentioned above, there is a growing awareness of the need for new ways of producing and consuming energy for more sustainable development [46,47]. The resulting CO_2_ does not increase the greenhouse effect because it comes from the CO_2_ contained in the atmosphere [48]. There seem to be many advantages to using renewable energy sources due to their availability and neutrality vis-à-vis pollution [49]. Sustainable economic growth requires a reliable and affordable energy supply. In many countries where biomass is the primary energy source, its use directly stimulates economic growth and provides energy for agriculture, industry, transportation, and services. Biomass, as a renewable energy source, also offers opportunities for local development. However, to realise the full economic potential of biomass, a robust legal framework is crucial. Therefore, the rule of law can ensure political stability, protect property rights, and enforce the law equitably, which is essential to encourage investments in energy infrastructure, ensure investment security, and promote a favorable business environment. Additionally, clear and consistent regulations regarding the sustainable use of biomass resources are necessary to prevent overexploitation, protect forest ecosystems, and ensure fair distribution of benefits among stakeholders. At the same time, government efficiency can also play a crucial role in promoting sustainable biomass use. An effective government is capable of formulating and implementing coherent and integrated energy policies, taking into account social, environmental, and economic aspects. This includes developing strategies for sustainable management of biomass resources, promoting clean and efficient energy technologies, and investing in research and development of innovative solutions. It is worth noting that transitioning to a biomass economy requires a comprehensive and inclusive approach, requiring close collaboration between government, businesses, civil society, and local communities. Stakeholder engagement at all levels is essential to address environmental sustainability challenges, energy security, and poverty reduction while harnessing biomass's economic opportunities. Economic growth, biomass energy consumption, the rule of law, and government efficiency are interdependent factors shaping sustainable development. Therefore, by adopting an integrated and collaborative approach, the country can fully leverage the potential of biomass to stimulate economic growth, promote social inclusion, and protect the environment for future generations [50]. The objective of this study is to determine the relationship between economic growth (GDP), biomass energy consumption (BEC), Rule of Law, and Government Effectiveness on climate change (CO2 emissions) in the Republic of Congo from 1990 to 2020. No prior empirical research has addressed this topic; it is still unexplored empirically, leaving a gap in the current literature. This study aims to fill the research gap with a rigorous and detailed approach. By venturing into uncharted territory, it hopes to shed light on new aspects and provide a unique perspective. As a result, this study makes significant contributions in several areas.

To begin, the choice of the Republic of Congo represents evident innovation and scientific progress, offering a unique perspective on a specific geographical context. This study fills a gap in the existing literature by examining the country's unique climate sustainability challenges and opportunities and identifying solutions tailored to local needs.

Another significant contribution of this study is to improve the methods section of the literature. This study uses a Nonlinear Autoregressive Distributed Lag (NARDL) analysis to investigate nonlinear effects among the variables. While other methods, such as Vector Autoregression, Multiple Linear Regression, and Structural Econometric Models, have been widely used in previous literature, the NARDL method introduces a new approach that allows for a more in-depth exploration of nonlinearity between economic and environmental variables, increasing its utility as a valuable tool for climate sustainability research.

Finally, the use of single-choice variables is an innovation in this study. To begin, economic growth recognises its critical role in policymaking and individual behaviour and its impact on consumption and production patterns that affect the climate. Second, in many developing countries, such as the Republic of Congo, biomass energy consumption is frequently underestimated but critical, with significant implications for greenhouse gas emissions and deforestation. Its inclusion provides a more comprehensive view of the country's environmental and climate challenges. Furthermore, analyses of the Rule of Law and Government Efficiency shed new light on how policies and institutions affect climate sustainability by influencing the implementation of environmental regulations and the ability to address climate challenges. By combining these variables, this study takes an innovative and comprehensive approach to better understanding the complex interactions between economic, environmental, and institutional factors influencing climate sustainability in the Republic of Congo. Finally, this multidimensional approach provides valuable insights for developing policies and strategies to promote climate resilience and sustainable development in the country. At the heart of this study are several critical research questions that will allow us to determine the relationship between economic growth (GDP), biomass energy consumption (BEC), the rule of law, and government effectiveness on climate change (CO2 emissions) in the Republic of Congo. Specifically, we will examine the following questions: How does economic growth affect greenhouse gas emissions in the Republic of Congo? How does biomass energy consumption impact climate change and environmental sustainability in the Congo? How much do the Rule of Law and Government Effectiveness influence initiatives to combat climate change and promote sustainable practices in the Republic of Congo? The article has been subdivided into five sections to answer our research questions. The Introduction section (1) allowed us to present the study's context and research objectives and justify its relevance. The Literature Review section (2) allowed us to examine and synthesise previous works on the subject, identifying gaps and explaining the need for the current study. The Materials and Methods section (3) describes the methodology and data analysis. The Empirical Results and Discussion section (4) presents and interprets the study's findings, emphasising key observations and discussing their implications in light of the research objectives and existing literature. The conclusion section (5) summarises the study's main findings, highlighting policy recommendations, policy implications, and limitations.

## 2 Literature review

### 2.1 GDP and CO2 emissions

Several authors have embarked on empirical studies on the link between GDP and CO_2_, which led them to draw several conclusions about this topic. Author Ang (2007) used an autoregressive distributed delay model (ARDL) between 1960 and 2000 and found an inverse relationship between CO_2_ emissions and economic growth. The author Gozgor [51] has worked on the relationship between GDP and CO_2_ in 35 OECD countries. The impact of financial development on the environment is based on a controversial assumption made by Grossman and Krueger called EKC (Environmental Kuznets Curve) [52].

Aqib Mujtaba [53]examines the symmetric (linear) and asymmetric (non-linear) effects of economic growth, capital formation, and renewable and non-renewable energy consumption on CO2 emissions and ecological footprint in 17 OECD countries from 1970 to 2016. The NARDL approach results show that economic growth has a positive (negative) impact on CO_2_. Ref [54] examines the relationship between economic growth and polluting emissions in Tunisia from 1961 to 2004. The results suggest a long-term relationship between carbon emissions and GDP. The results of the PMG estimation technique reveal that the relationship between CO_2_ emissions is U-shaped.

Refs [[55], [56], [57], [58]], in their research, find inverted U-shaped relationships between GDP and CO2, while [[59], [60], [61]] find a constantly increasing curve relationship between GDP and CO_2_, on the other side [62,63] revealed a U-shaped curvature. Other studies, such as [[64], [65], [66]], have found negligible associations between economic growth and environmental degradation. Regarding the asymmetric (non-linear) relationship between economic growth and CO_2_ emissions, for example, AhAtil [67] analysed data from Q1 1970 to Q4 2015 in China and found the asymmetric effect of economic growth on CO_2_ emissions. Responding to the analysis of the NARDL approach, the results show that positive changes in economic growth had positive effects on CO_2_ emissions and that negative changes in economic growth had negative effects on CO_2_ emissions. refs [68] worked in Saudi Arabia from 1971 to 2014 and applied the NARDL model to conclude that positive and negative changes in economic growth have a positive impact on CO_2_ emissions. In their research in Saudi Arabia between 1990 and 2014, researchers [69], using the NARDL model, showed that positive and negative changes in economic growth had no significant effect on CO_2_ emissions. Refs [[70], [71], [72]] found that positive effects in economic growth have a positive shock on CO_2_ emissions, and negative shocks in economic growth negatively impact CO_2_ emissions. However, the link between economic growth and CO_2_ emissions is a link in the form of an inverted U, which validates the environmental Kuznets curve (EKC). Litavcová [73] studied the long-term and short-term dynamics between carbon emissions, energy consumption, and economic growth and the exploitation of causality in 14 countries in the Danube region between 1990 and 2019. The results show a long-term relationship between carbon emissions, energy consumption, and economic growth. Refs [74] focused on Environmental Sustainability in India and found that corruption, environmental dangers, GDP, and urbanisation positively influence India's carbon emissions. The empirical results of long-term estimates show that for every 1 % increase in GI, GDP, and D&F industries, carbon dioxide emissions increase by −0.079 %, 0.566 %, and 0.143 %, respectively [75].

By seeking to understand how financial developments, increased primary energy consumption, and technological innovations affect the prospects of BRICS countries to achieve economic and environmental sustainability, the findings of the study [76]show that there is a modest link between the variables that make it possible to achieve this economic and environmental sustainability. Another benchmark [77]assesses the non-linear (asymmetric) impact of natural gas consumption on renewable energy consumption and economic growth in Saudi Arabia and the United Arab Emirates, as well as other consumption drivers such as trade openness and CO2 emissions. The results confirm the long-term association between the variables for Saudi Arabia and the United Arab Emirates.

### 2.2 Biomass and CO2 emissions

Biomass is a product made from self-renewable natural resources. Many governments have made biomass energy one of their main objectives to reduce climate degradation and demonstrate that biomass energy consumption will provide the means for sustainable development and a green economy [[78], [79], [80], [81]]. The author [[82], [83], [84]] explores the relationship between renewable energy consumption and climate change, claiming that biomass energy consumption can significantly affect the climate as it can alter a country's ecological footprint.

In their research, the authors [67,[85], [86], [87], [88], [89], [90], [91]] found that biomass energy consumption negatively predicted ecological footprint and CO2 emissions. Cointegration was analysed using an autoregressive distributed delay (ARDL) model. Next, Johansen's cointegration technique is applied by adding intermittent variables from 1977 to 2011 in China. The results affirm a long-term, two-way link between renewable energy consumption and economic growth [92].

KISHORE [93] found that biomass energy significantly reduced carbon emissions. The conclusions of OLABI [94] align with those of [95,96], which also found a link between the consumption of renewable energy, biomass, and climate change or carbon emissions. refs [97] analyse the environmental impact of biomass energy, financial development, and economic growth on annual data for 1965–2018 in the United States using an Autoregressive Distributed Fourier Lag (ARDL) method. The results prove that biomass improves the quality of the environment. Other researchers, for example [98,99], found that biomass and renewable energy can help minimise climate breakdown. For the researcher [100], biomass energy impacts reducing carbon dioxide emissions. Katircioglu [101] also supports this and applies the ARDL limit test method to Turkey, concluding that biomass can reduce CO_2_ emissions. Dogan [102] found a biomass-reducing effect on CO_2_ emissions by applying LM panel bootstrap cointegration tests and FMOLS methods in 22 countries. Muhammad Shahbaz [103] analysed EKC hypotheses across bulk renewables and trade in the United States. The analysis shows that the link between growth and CO_2_ emissions is U-shaped and inverted N-shaped, with discontinuities. Biomass consumption reduces CO_2_ emissions. Author Sarkody [104]applied the ARDL approach and concluded that biomass can reduce greenhouse gas emissions in Australia. Researcher Balezentis [105] conducted panel, FMOLS, and DOLS fixed effects analyses in 27 EU countries. They found that biomass energy is the best option to minimise the carbon impact compared to other types of renewable energy. Refs [106] study the relationship between biomass energy, real income, and CO_2_ emissions in China. The analyses are estimated using a Dynamic Autoregressive Distributed Lag (DARDL) model for 1982–2017. The results showed that biomass energy consumption was negatively correlated with China's CO2 emissions, showing how biomass energy consumption could be a solution to reduce pollution. Gyamfi [107] conducted four panel tests in seven emerging countries and found that biomass significantly reduces CO_2_ emissions. Researchers [[108], [109], [110]] applied several methods and concluded that biomass energy consumption significantly reduced CO2 emissions in Thailand, Germany, and others. Renewables have become more coveted than any other energy source. In their analysis of the relationship between biomass energy consumption and CO_2_ emissions in the top 10 biomass energy consuming countries (Brazil, Canada, Thailand, China, Italy, India, Germany, United States, United Kingdom, and Japan) [111], in an approach "Quantile on quantile (QQ) data from 1991 to 2018, the author argues that, except for Thailand, biomass energy consumption typically reductions in CO_2_ emissions for different quantiles of selected countries.

Contrary to many findings, Ahmed [112] examines the causal relationship between biomass energy use and carbon emissions using the PARDL method and claims that biomass energy use reduces carbon emissions but not significantly. The authors confirm that this insignificance is due to the low share of biomass energy in total energy in these countries. In addition, authors [113] focused on West Africa by applying the least squares triple. Their results reported the appropriate role of biomass in three countries, while this renewable energy source increased pollution in five other countries. Solarin [114] took the same direction, using a system generalised method of moments in 80 countries, confirming that biomass consumption produces more CO_2_ emissions. Mahmood [115] focuses on the ARDL method in Pakistan and finds that biomass increases CO_2_ emissions. Shahbaz [116] used the GMM approach to conclude that biomass consumption produces CO_2_ emissions in G7 countries. Gao [117] used Kao's panel cointegration test and FMOLS estimators in 13 Asian countries and found that biomass leads to more CO_2_ emissions. The authors [118,119] study the impact of non-renewable and renewable energy consumption on economic growth and ecological footprint. They conclude that biomass increases CO_2_ emissions. Kim [120] examined the relationship between monthly carbon emissions and biomass energy use in the United States from 1973 to 2013. The results show biomass energy can reduce carbon emissions in the United States. In addition, they add that energy policy should encourage increased biomass production to reduce emission levels. Author Sinha [121] found in their studies that biomass energy increases carbon emissions and harms the environment. Refs [122] examines the interaction between biomass energy and carbon emissions in the United States. Wavelet coherence techniques led him to conclude that in the short term (period of 1–4 years), the carbon impact of biomass energy use from 1984 to 2005 was positive; however, in the longer term (4–8-year period), use minimising emissions levels in the United States was negatively correlated between 2006 and 2015. Md Shabbir Alam [84] studied Empirical analysis for the period 1980–2020 using Autoregressive Distributed Lag (ARDL) bounds tests and Vector Error Correction Model (VECM) Granger causal cointegration. The analysis does not suggest a short-term causal relationship between carbon emissions, energy consumption, and other variables of their study, but rather a long-term relationship between exogenous and endogenous variables. refs [123] examined the relationship between renewable energy consumption and atmospheric quality in India and found an inverted U-shape.

### 2.3 Rule of law, government effectiveness and CO_2_ emissions

The debate on environmental, climate impact and law is intensifying as companies are increasingly willing to create a supportive set of rules and capitalise on this incremental growth [124]. Climate management should cooperate with the rule of law to reduce CO_2_ emissions and improve the quality of the environment. In our research, the rule of law means that a country's institutions, businesses, and government follow a structured legal framework to maintain order. In other words, a good rule of law system contributes to a country's economic and social development. In addition, it can help control the progressive deterioration of environmental conditions by applying environmental standards. Refs [125] examined the effect of governance quality on pollution as a modified form of the environmental Kuznets curve. Using the cross-sectional method and cross-sectional heterogeneity, using panel data, it was found that, even in countries with political stability and effective governance, the absence of appropriate environmental laws, regulations, and policies can contribute to pollution. The authors encourage a better legislative framework for pollution control. Ref [50]shows that managers' personality influences the adoption and acceptance of decisions to improve their work environment. According to Ref. [126], the rule of law is essential to identify the activities to be carried out, determine the necessary means, program the realisation of the activities, and improve taxation for climate purposes. For researchers [127,128], the rule of law indicates the level of knowledge of the applicable laws of a country or society, including policies and regulations. The author [129] states that it is an institutional indicator that uniquely improves environmental conditions. A strong rule of law ensures the adaptability of policies and encourages companies to comply with directives related to environmental stability. It also gives a feeling of respect for anti-pollution protocols [130]. According to the author [131], a stable rule of law helps limit carbon emissions and increase economic growth. For the author [132], the rule of law has a negative impact on the environment. Researcher [133] shows that the rule of law is essential to limit carbon emissions and improve the quality of the environment. According to Ref. [134], the correlation between political stability and carbon emissions is found that political stability contributes positively to environmental stability. Researchers [135] also arrived at the same result and concluded that political stability can reduce carbon emissions and contribute to good environmental health. Ref [136]finds that strong institutions are essential to address renewable energy development and reduce carbon emissions in the long term. Innovation requires robust systems of law and quality institutions to establish the continuity of these innovation policies. Companies are looking for innovative technologies that match their investments and development. Without a strong system of law, innovation will struggle to thrive and contribute to economic and environmental sustainability [137]. There is a relationship between government effectiveness and CO2. Governance is multidimensional and can be defined through several indicators recognised by legislation, each focusing on a specific role while helping to determine public choices and occupying an important place in the application and compliance with regulations, improving the quality of the environment [4]. Refs [24,25]examined social media and innovation in decision-making. The promotion of renewable energies and the fight against climate change are made possible by innovation and networks. They can help dispel false information and create a sustainable future when used wisely. Therefore, the sustainability of energy resources and environmental protection interact directly and indirectly with institutional quality.

## 3 Materials and methods

### 3.1 Data and variables

This study aims to examine the asymmetric relationship between biomass energy consumption, economic growth, and the Rule of law on CO_2_ in the Republic of Congo using a nonlinear ARDL approach. Data for the study came from World Development Indicators, Worldwide Governance Indicators, and the International Resource Panel. This study uses annual data from 1990 to 2020 of variables Economic Growth (proxy Gross Domestic Product (GDP), Biomass Energy Consumption (BEC), Rule of Law (RL), and government effectiveness (GE) on Climate change (proxy CO2 emissions in kilo tons). Economic growth is GDP in constant 2015 US$, Biomass Energy Consumption is the Domestic Material Consumption (t), Rule of Law and Government Effectiveness are a measure of the quality of governance with values ranging from 2.5 (weak) to 2.5 (strong), Climate change is CO2 emissions (metric tons per capita). A detailed description of the variables is listed in Table 1. The Republic of Congo has warm temperatures and abundant rainfall in the equatorial region. It is particularly vulnerable to the effects of climate change. One of the leading causes of greenhouse gas emissions in the Republic of Congo is deforestation, exacerbated by these climate conditions. Multiple factors, including biomass energy consumption, government efficiency, economic growth, and the rule of law, influence climate change in the Republic of Congo. Economic growth can have positive or negative impacts on climate change. Since an increase in energy consumption often accompanies economic growth, it may increase greenhouse gas emissions. On the other hand, economic growth can also help reduce greenhouse gas emissions by encouraging investment in cleaner, more efficient technologies. Effective governments can have a positive impact on climate change. Effective governments can implement effective climate policies to reduce greenhouse gas emissions. Positive impacts on climate change can also be attributed to the rule of law. A strong constitutional state can ensure that climate policies are implemented fairly and effectively. In the Republic of Congo, biomass energy significantly impacts climate change. These variables have been examined in the literature on climate change in different ways. Several studies have looked into the connection, from a general functional standpoint, between these variables and CO2 emissions. The connection between these variables and the consequences of CO2 emissions in particular settings, such as developing and underdeveloped nations, has been examined in another research [111,124,125,133,138].

**Table 1:** Data and variables.

Variables description	Variables Code	Unit of Measurement	Data Sources
CO2 emissions/Climate Change	CO2	CO2 emissions (metric tons per capita)	World Development Indicators (WDI 2022) https://databank.worldbank.org/source/world-development-indicators (accessed on September 6, 2023)
Economic Growth/Gross Domestic Product	GDP	GDP (constant 2015 US$)	World Development Indicators (WDI 2022) https://databank.worldbank.org/source/world-development-indicators (accessed on September 6, 2023)
Biomass Energy Consumption	BEC	Domestic Material Consumption (t)	International Resource Panel https://www.resourcepanel.org/global-material-flows-database (accessed on September 6, 2023
Rule of Law	RL	A measure of the quality of governance with values ranging from 2.5 (weak) to 2.5 (strong)	Worldwide Governance Indicators (WGI 2022) https://databank.worldbank.org/source/worldwide-governance-indicators (accessed on September 6, 2023)
Government Effectiveness	GE	A measure of the quality of governance with values ranging from 2.5 (weak) to 2.5 (strong)	Worldwide Governance Indicators (WGI 2022) https://databank.worldbank.org/source/worldwide-governance-indicators (accessed on September 6, 2023)

This study could also examine other variables. For example, we could research the impact of climate policy, green technology, or individual behaviour on climate change in Congo. Nonetheless, the significance of the four variables under investigation stems from their close association with climate change. Economic growth, government efficiency, rule of law, and biomass energy consumption influence greenhouse gas emissions. Therefore, studying these four variables can reveal crucial details regarding the reasons behind Congo's climate change and its actions and strategies to counter it. Knowing how these factors influence climate change in Congo is essential for several reasons. First, it could clarify the causes of climate change in Congo. Second, it can help identify feasible plans and actions to reduce greenhouse gas emissions and combat climate change. The application of the nonlinear ARDL method of Shin et al. (2014) is innovative in this area. This approach accounts for nonlinear relationships between variables critical to nonlinear phenomena such as climate change. This approach helps to elucidate the relationship between the variables studied and climate change in Congo. This can lead to more successful climate actions and interventions.

This research builds on the robust model developed by Shin [139] to investigate the relationship between economic growth variables, government effectiveness, rule of law, and biomass energy consumption on climate change. The Shin [139] nonlinear ARDL method is a significant statistical technique that can be applied to examine the connection between economic factors and climate change. It is comparatively easy to use, robust, and capable of testing for the existence of nonlinear relationships. It is utilised, for instance, to research the connections between energy costs and climate change, greenhouse gas emissions and economic growth, and climate policy and climate change [97,138]. Given the objectives of this research, the following equation will be studied to test the relationship between climate change represented by CO2 and other variables.

(1) $$\begin{array}{c} CO2=ƒ\;\left(GDP,BEC,RL,GE\right) \end{array}$$

CO2 is the representative variable of climate change, GDP is the variable measuring economic growth, BEC is the energy source proposed to limit carbon impact in the Republic of Congo, and RL and GE measure the policy-maker because researchers need concrete measures of government quality to determine the effect of the latter, in particular with economic development and environmental stability. Our choice is close to the results of economic theory and empirical research by the authors [140], which states that there are several reasons to think that time series, economic, environmental, or financial, can follow non-linear trajectories. This is mainly due to the endogenous and exogenous nature of the variables. Therefore, more appropriate models must be applied to reflect real-world complexity accurately. It is in this context that, for the analysis of this article, we will adopt the NARDL model developed by Shin [139]. Shin et al. explained that the research revolves around cointegration and non-stationarity analysis. Dickey and Fuller, Engel and Granger, Johansen, Phillips and Hansen, and Kwiatkowski confirm the same. In traditional cointegration analysis, all variables can be non-stationary. The NARDL model uses a boundary-testing approach to test long-term and short-term stable relationships. Asymmetric cumulative dynamic multipliers are then derived, allowing the display of asymmetric fit curves for explanatory variables after positive and negative shocks. This evaluation strategy aims to: a) examine the positive and negative effects of the decomposed variable on the dependent variable when the dependent variable shows positive and negative changes. b) The model is flexible because it is not necessary to integrate the variables in the same order. c) It is a dynamic representation that provides robust empirical results even for small sample sizes. Using NARDL is appropriate because it captures the varied and non-linear links between economic and environmental variables, including economic growth, biomass energy consumption, the rule of law, and government effectiveness regarding climate change in the Republic of Congo. Unlike more traditional methodologies that assume linear relationships between variables, NARDL allows for more subtle dynamics and the detection of asymmetric and lagged effects, which is critical in the complex context of climate change.

In addition, the NARDL model has specific benefits over various approaches, particularly its ability to account for both short-term and long-term effects of explanatory variables on the dependent variable. This is especially important in our research, where we want to know how economic growth and government effectiveness affect climate sustainability in the long run while also considering short-term adjustments.

Regarding the implications for our results, the NARDL model enables us to obtain robust and precise estimates of the relationships between our variables of interest while accounting for their complex and dynamic interactions. This allows us to identify the underlying mechanisms that govern the interactions of economic growth, biomass energy consumption, the rule of law, government effectiveness, and their impact on climate sustainability in the Republic of Congo. Finally, using the NARDL model improves the validity and reliability of our findings while also providing valuable insights for policymakers seeking to promote sustainable and climate-resilient development in the country. In our study, we applied the Augmented Dickey-Fuller Test (ADF) [141] and Phillips Perron (PP) [142] for unitary roots. Our study also used the Wald test to test for nonlinearity.

Therefore, equation (1) can be rewritten as follows:

(2) $${lnCO2}_{t}=\alpha_{t}+{\sigma_{t}+\beta }^{+}{lnGDP}_{t}^{+}+\beta^{-}\;{linGDP}_{t}^{-}+\beta^{+}\;{linBEC}_{t}^{+}+\beta^{-}{linBEC}_{t}^{-}+\beta^{+}{linRL}_{t}^{+}+\beta^{-}{linRL}_{t}^{-}+\beta^{+}{linGE}_{t}^{+}+\beta^{-}{linGE}_{t}^{-}+u_{t}$$

Where lnCO2, lnGPD, lnBEC, lnRL, and lnGE are the natural logarithms of CO2, GDP, BEC, RL, and GE, respectively. Converting variables to their natural logarithm form reduces data skew. α is the point of intersection, σ presents the trend effect, β is the variable's coefficient, u is the error term, and t represents time. Therefore, the nonlinear auto-regressive distributed lag (NARDL), equation (2) can be written in the form of equation (3):

(3) $${\Delta lnCO2}_{t}={\mu +\forall lnCO2}_{t-1}+\pi^{+}{lnGDP}_{t-1}^{+}+\pi^{-}{lnGDP}_{t-1}^{-}+\emptyset^{+}{lnBEC}_{t-1}^{+}+\emptyset^{+}{lnBEC}_{t-1}^{-}+\propto^{+}{lnRL}_{t-1}^{+}+\propto^{-}{lnRL}_{t-1}^{-}+\cup^{+}{lnGE}_{t-1}^{+}+\cup^{-}{lnGE}_{t-1}^{-}+\emptyset^{+}{lnBEC}_{t-1}^{+}+\emptyset^{+}{lnBEC}_{t-1}^{-}+\propto^{+}{lnRL}_{t-1}^{+}+\propto^{-}{lnRL}_{t-1}^{-}+\cup^{+}{lnGE}_{t-1}^{+}+\cup^{-}{lnGE}_{t-1}^{-}+\sum_{i=0}^{v3}\left(\emptyset^{+}{lnBEC}_{t-1}^{+}+\emptyset^{+}{lnBEC}_{t-1}^{-}\right)+\sum_{i=0}^{v4}\left(\propto^{+}{lnRL}_{t-1}^{+}+\propto^{-}{lnRL}_{t-1}^{-}\right)+\sum_{i=0}^{v5}\left(\cup^{+}{lnGE}_{t-1}^{+}+\cup^{-}{lnGE}_{t-1}^{-}\right)+\varepsilon_{t}$$

The short-term NARDL resilience with an error correction mechanism can be estimated as equation (4).

(4) $${\Delta lnCO2}_{t}=\mu +\sum_{i=1}^{v-1}\alpha_{i}{\Delta lnCO2}_{t-i}+\sum_{i=0}^{v2}{(\pi }_{i}^{+}{\nabla lnGDP}_{t-i}^{+}+\pi_{i}^{-}{\nabla lnGDP}_{t-i}^{-})+\sum_{i=0}^{v3}\left(\emptyset^{+}{lnBEC}_{t-1}^{+}+\emptyset^{+}{lnBEC}_{t-1}^{-}\right)+\sum_{i=0}^{v4}\left(\propto^{+}{lnRL}_{t-1}^{+}+\propto^{-}{lnRL}_{t-1}^{-}\right)+\sum_{i=0}^{v5}\left(\cup^{+}{lnGE}_{t-1}^{+}+\cup^{-}{lnGE}_{t-1}^{-}\right)+{\psi ECM}_{t-1}+{\;\varepsilon }_{t\;}$$

As indicated in equation (2), the effect of the variables GDP, BEC, RL, and GE can be subdivided into two (positive change and negative change), as shown in equations (5), (6), (7), (8)).

(5) lnBECΟ+lnBECt++lnGDPt−

(6) lnGDPΟ+lnGDPt++lnBECt−

(7) lnRLΟ+lnRLt++lnRLt−

(8) lnRLΟ+lnGEt++lnGEt−

Where ${lnGDP}_{Ο}\text{,}$ ${lnBEC}_{Ο}$, ${lnRFL}_{Ο}$ and ${lnGE}_{Ο}$, represent the random initial value, ${lnGDP}_{t}^{+}+{lnGDP}_{t}^{-},{lnBEC}_{t}^{+}+{lnBEC}_{t}^{-}\text{,}$ ${lnRL}_{t}^{+}+{lnRL}_{t}^{-}$ and ${lnGE}_{t}^{+}+{lnGE}_{t}^{-}$ are variables represent the fraction and the process of cumulative positive and negative change, respectively as shown in equations (10), (11), (12), (13), (9)).

(9) $$\begin{array}{c} {lnGDP}_{t}^{+}=\sum_{i=1}^{t}{\Delta lnGDP}_{i}^{+}=\max\left({\Delta lnGDP}_{i},Ο\right),{lnGDP}_{t}^{-}=\sum_{i=1}^{t}\min\;\left({\Delta lnGDP}_{i},Ο\right)+\varepsilon_{t\;} \end{array}$$

(10) $$\begin{array}{c} {lnBEC}_{t}^{+}=\sum_{i=1}^{t}{\Delta lnBEC}_{i}^{+}=\max\left({\Delta lnBEC}_{i},Ο\right),{lnBEC}_{t}^{-}=\sum_{i=1}^{t}\min\;\left({\Delta lnBEC}_{i},Ο\right)+\varepsilon_{t\;} \end{array}$$

(11) $$\begin{array}{c} {lnRL}_{t}^{+}=\sum_{i=1}^{t}{\Delta lnRL}_{i}^{+}=\max\left({\Delta lnRL}_{i},Ο\right),{lnRL}_{t}^{-}=\sum_{i=1}^{t}\min\;\left({\Delta lnRL}_{i},Ο\right)+\varepsilon_{t\;} \end{array}$$

(12) $$\begin{array}{c} {lnGE}_{t}^{+}=\sum_{i=1}^{t}{\Delta lnGE}_{i}^{+}=\max\left({\Delta lnGE}_{i},Ο\right),{lnGE}_{t}^{-}=\sum_{i=1}^{t}\min\;\left({\Delta lnGE}_{i},Ο\right)+\varepsilon_{t\;} \end{array}$$

To test the long-term symmetry and asymmetry an estimation $\theta^{+}=\theta^{-}\theta^{+}\neq \theta^{-}$ of the asymmetric cumulative dynamic multipliers impact of the variables can be constructed by performing an approximate Wald test as demonstrated in equation (13).

(13) $$\begin{array}{c} p_{n}^{+}=\sum_{i=0}^{n}\frac{\sigma {lnCO2}_{t+i}}{{\sigma lnGDP}_{t-1}^{+}},p_{n}^{-}=\sum_{i=0}^{n}\frac{\sigma {lnCO2}_{t+i}}{{\sigma lnGDP}_{t-1}^{-}},p_{n}^{+}=\sum_{i=0}^{n}\frac{\sigma {lnCO2}_{t+i}}{{\sigma lnBEC}_{t-1}^{+}},\\ p_{n}^{-}=\sum_{i=0}^{n}\frac{\sigma {lnCO2}_{t+i}}{{\sigma lnBEC}_{t-1}^{-}}{,p}_{n}^{+}=\sum_{i=0}^{n}\frac{\sigma {lnCO2}_{t+i}}{{\sigma lnRL}_{t-1}^{+}},p_{n}^{-}=\sum_{i=0}^{n}\frac{\sigma {lnCO2}_{t+i}}{{\sigma lnRL}_{t-1}^{-}}\text{,}\\ {\;p}_{n}^{+}=\sum_{i=0}^{n}\frac{\sigma {lnCO2}_{t+i}}{{\sigma lnGE}_{t-1}^{+}},p_{n}^{-}=\sum_{i=0}^{n}\frac{\sigma {lnCO2}_{t+i}}{{\sigma lnGE}_{t-1}^{-}}n=0,1,2\ldots \; \end{array}$$

## 4 Empirical result and discussion

### 4.1 Result

The analysis of our NARDL model follows eight steps: the 1 step is to carry out descriptive statistics, which makes it possible to see if the sample is representative, to estimate the parameters of the observed data to analyse better the following statistics: mean, median, variance, standard deviation, quantile. The 2 examines correlation, which gives an idea of the degree of association or covariation between two variables. The 3 step checks the stationary properties of each variable using a unit root test, which defines the order in which the variables are integrated by the Augmented Dickey-Fuller (ADF) and Phillips Perron (PP) stationarity tests. The 4-step cointegration test results to analyse the long-run and short-run relationship between the above variables on CO_2_ emission. The 5-step Wald Test checks if there is a long-term relationship between the variables and whether the long-term relationship is significant. The 6-step diagnostics test results are as follows: Jarque-Bera, Breusch-Godfrey serial correlation LM, Heteroskedasticity Test: Breusch-Pagan-Godfrey, Ramsey Reset. The 7-step NARDL model was used to see the positive and negative GDP, BEC, RL, and GE shocks on CO2 emission. Finally, the 8-step Stability diagnostic and dynamic multiplier diagrams were used.

#### 4.1.1 Descriptive statistic

We examined annual data for Congo for the period 1990–2020. Based on our objectives, the experimental parameters or variables designed for the article are as follows: Climate change, represented by carbon dioxide emissions (CO2), is an endogenous variable in this study. Despite these shortcomings, this study considers energy management to measure the Republic of Congo's climate stability. This indicator allows us to have an overview of the possible relationship between renewable energy consumption represented by biomass energy consumption (BEC), economic growth (GDP), rule of law (RL), and government effeteness (GE). Descriptive statistics for all variables are presented in Table 2. The log yields of the different variables vary considerably. The most comprehensive range of variation was observed in the GDP. These higher scatterplots indicate that GDP can play a crucial role in climate sustainability and economic development in the Republic of Congo. As can be seen from these results, the coefficient of variation, or relative standard deviation statistic, is significantly lower in logarithmic terms, highlighting the low dispersion around its mean. Therefore, we can say that the variances of CO_2_, BEC, GDP, ER, and GE are slightly skewed to the right after statistical analysis of asymmetry and flattening. From this descriptive statistical analysis above, we can infer that there is strong evidence to reject the null hypothesis that data from all variables are normally distributed. The asymmetry value must be 0 for normal distribution and 3 for flattening, which allows us to employ the correlation test**.**

**Table 2:** Descriptive statistic.

	Mean	Median	Maximum	Minimum	Std. Dev.	Skewness	Kurtosis	Positive(+) sums of variables	Negative(−) sums of variables
LNCO2	−0.634	−0.647	−0.235	−0.985	0.171	0.248	2.937		
LNGDP	22.785	22.786	23.235	22.429	0.267	0.181	1.585	−0.739	−1.449
LNBEC	−0.537	0.147	0.394	−2.302	1.150	−0.912	1.849	−1.145	0.086
LNRL	−0.944	−0.714	0.462	−2.302	0.993	−0.308	1.642	0.091	−0.036
LNGE	−0.509	0.185	0.460	−2.302	1.169	−0.906	1.847	0.108	−0.484

LNCO2: Climate Change; LNGDP: Economic Growth; LNBEC: Biomass Energy Consumption; LNLR: Rule of Law and LNGE: Government Effectiveness.

#### 4.1.2 Correlation

After checking the descriptive statistics for the variables used in our study, we are now checking the correlation between variables. The Bravais-Pearson correlation coefficient is a statistical measure that indicates the strength and direction among dual variables. Therefore, it is a crucial factor in our results. Consequently, the closer the coefficient value is to +1 or −1, the stronger the strength of the liaison, and the closer it is to 0, the weaker it is. Thus, any increase at the level of A corresponds to the rise at the level of B (positive correlation). Any increase at the level of A corresponds to a decrease at the level of B (negative correlation). Except for the GDP variable, which negatively impacts the CO2 variable, the rest of the variables in Table 3 show a significant positive link between CO2 in the Republic of Congo with a coefficient of −0.058, 0.054, 0.073, and 0.065, respectively. Indeed, the negative impact of GDP variables on CO_2_ indicates that when economic growth increases, CO_2_ decreases in the Republic of Congo. The results further show that BEC, RL, and GE significantly impact the GDP with a positive coefficient of 0.709, 0.379, and 0.730, respectively. RL and GE positively impact the BEC with a coefficient of 0.890 and 0.997, while GE positively impacts RL with a coefficient of 0.874. Negative correlations between variables suggest that other factors influence this relationship between variables.

**Table 3:** Correlation test result.

	LNCO2	LNGDP	LNBEC	LNRL	LNGE	Positive(+) sums of variables	Negative(−) sums of variables
LNCO2	1						
LNGDP	−0.058	1				−0.739	−1.449
LNBEC	0.054	0.709	1			−1.145	0.086
LNRL	0.073	0.379	0.890	1		0.091	−0.036
LNGE	0.065	0.730	0.997	0.874	1	0.108	−0.484

LNCO2: Climate Change; LNGDP: Economic Growth; LNBEC: Biomass Energy Consumption; LNLR: Rule of Law and LNGE: Government Effectiveness.

#### 4.1.3 Unit root test

Since the correlation between variables has been proven, it is pertinent to check the stationary time series among variables by running the unit root test. The results of the augmented stationarity tests of Dickey-Fuller and Phillips Perron are presented in Table 4 according to the findings of the ADF test [141] and Phillips-Perron [142]. The findings show that CO_2_, GDP, RL, and GE are non-stationary at level, but they become stationary after the first difference, while BEC is stationary at level in both tests and RL in (PP) test. Most are integrals in order (1), except the variable BEC and RL, which is an integral of order (0); none of them are stationary at the second difference, allowing us to continue with the NARDL model. However, before processing with the NARDL model and determining the order of integration of the different variables, it is necessary to check out the cointegration bound test to determine the existence of long-run relationships between the variables (see Table 5).

**Table 4:** Unit root test result.

	Level		First Difference		Order of Integration
Augmented Dickey-Fuller test statistic result					
LNCO2	−1.691	−1.780***	−4.700^a^	−4.805^a^	I (1)
LNGDP	−1.172	−0.193	−3.820^a^	−3.925^a^	I (1)
LNBEC	−1.655^a^	−1.725^a^	−19.234^a^	−19.179^a^	I (0)
LNRL	−2.064	−2.058**	−16.893^a^	−16.660^a^	I (1)
LNGE	−1.664	−1.804	−19.493^a^	−19.459^a^	I (1)
Phillips-Perron test statistic result					
LNCO2	−1.691	−1.999	−4.673^a^	−4.771^a^	I (1)
LNGDP	−1.189	−0.859***	−3.842^a^	−3.903	I (1)
LNBEC	−3.250**	−6.384^a^	−24.634^a^	−39.204^a^	I (0)
LNRL	−4.683^a^	−5.594^a^	−18.020^a^	−19.430^a^	I (0)
LNGE	−3.428	−6.753	−28.354^a^	−54.475^a^	I (1)

a , Means significant at 1 % and ** means significant at 5 %; LNCO2: Climate Change; LNGDP: Economic Growth; LNBEC: Biomass Energy Consumption; LNLR: Rule of Law and LNGE: Government Effectiveness.

**Table 5:** NARDL cointegration test result.

Test Statistic	Value	K
F-statistic	5.740	8
Critical Value Bounds		
Significance	I(0) Bound	I(1) Bound
10 %	1.85	2.85
5 %	2.11	3.15
2.5 %	2.33	3.42
1 %	2.62	3.77

#### 4.1.4 cointegration test result

The bound test analyses the long-run and short-run relationship between the above variables on CO2. The findings reported in Table (5) show that the calculated F-statistic value is 5.740, above the upper and lower limits of the bound test. The upper critical value is 3.77 at a 1 % significance level, meaning the null hypothesis of no cointegration relationship between LNGDP, LNBEC LNRL, LNGE, and LNCO_2_ is rejected. This means a long-run relationship exists between the selected variables and LNCO_2_. This result, allowing us to estimate the non-linear ADRL model, supports the bounds test result that shows a cointegration among the variables in the long run.

#### 4.1.5 Wald Test

Looking at the results in Table 6, we see a long-term relationship between the variables. The Wald criteria say the null hypothesis is rejected if the calculated statistic F exceeds the upper critical value I (1). The null hypothesis cannot be rejected if the statistic F is less than the lower critical value I (0). The result is considered indeterminate when it is between I (0) and I (1). Therefore, the variables will have an asymmetric relationship, meaning a long-term relationship exists between the variables studied [143]. Our results confirm the long-term skewed relationship between the variables. Thus, the null hypothesis must be rejected, and a long-term relationship between the variables in the model must be concluded. It can be inferred that economic growth, biomass energy consumption, economic growth rule of law, and government effectiveness have significant non-linear relationships with climate change and the selected variables in the Republic of Congo.

**Table 6:** Wald test results.

Wald test result	
Long-run asymmetry	
LNGDP	7.367*
LNBEC	6.962*
LNRL	0.677**
LNGE	8.767*
Short-run asymmetry	
LNGDP	0.323**
LNBEC	0.921***
LNGE	14.837*

LNCO2: Climate Change; LNGDP: Economic Growth; LNBEC: Biomass Energy Consumption; LNLR: Rule of Law and LNGE: Government Effectiveness.

##### 4.1.5.1 Diagnostics test results

After confirming the cointegration, the diagnostic checks are utilised in Table 7. Based on the Jarque-Bera, the residuals of the test are normal. The value is 0.271, and the probability (0.873) is greater than 0.05. The Breusch-Godfrey serial correlation LM test proves that the residual obtained is free from serial correlation. The Obs*R-squared is 3.399, and the probability is 0.182. Heteroskedasticity Test: Breusch-Pagan-Godfrey proves that the residuals obtained are free from Heteroskedasticity. The Obs*R-squared is 10.042, and the probability is 0.186, greater than 0.05. Finally, the Ramsey Reset test was used to check the appropriate functional form. The probability value of the F-statistic is 0.064, suggesting that the model is well specified. The model's performance for all the tests is entirely successful, and based on these findings, we can apply for the NARDL model.

**Table 7:** Diagnostics test results.

Test	Jarque-Bera	Breusch-Godfrey serial correlation LM	Heteroskedasticity Test: Breusch-Pagan-Godfrey	Ramsey Reset
Test Statistics	0.271	3.399	10.042	3.812
Probability	0.873	0.182	0.186	0.064

After confirming the stationary and the cointegration among the variables, we can run the NARDL model to see the positive and negative shocks of GDP, BEC, RL, and GE on CO_2_ in the Republic of Congo. Table 8 presents the long-run and short-run results. The optimal number of delays was selected according to the Akaike Information Criterion (AIC). The NARDL (1, 0, 1, 0, 1, 0, 0, 1, 0) model is best based on the Akaike information criterion (AIC) and various diagnostic tests. The table below explains the variables' positive and negative shocks in the long-run and short-run.

**Table 8:** NARDL test result.

Variable	Coefficient	Std. Error	t-Statistic	Prob. *
Long-run				
LNCO2(-1)	0.514	0.173	2.965	0.009
LNGDP_POS	−0.739	0.301	−2.459	0.025
LNGDP_NEG (−1)	−1.449	0.773	−1.874	0.059
LNBEC_POS	−1.145	0.315	−3.636	0.002
LNBEC_NEG (−1)	0.086	0.041	2.115	0.050
LNRL_POS	0.091	0.081	1.128	0.276
LNRL_NEG	−0.036	0.098	−0.374	0.713
LNGE_POS (−1)	0.108	0.040	2.719	0.015
LNGE_NEG	−0.484	0.653	−0.741	0.469
C	−0.379	0.136	−2.779	0.013
Short-run				
D(LNGDP_NEG)	0.374	0.657	0.568	0.577
D(LNBEC_NEG)	0.656	0.684	0.959	0.351
D(LNGE_POS)	1.102	0.286	3.852	0.001

LNCO2: Climate Change; LNGDP: Economic Growth; LNBEC: Biomass Energy Consumption; LNLR: Rule of Law and LNGE: Government Effectiveness; POS: POSITIVE; NEG: NEGATIVE.

Table 8 presents the estimation of the analysis of the long-run and short-run estimates of the NARDL model. The objective is to verify the nonlinear link between the variables GDP, BEC, RL, GE, and CO_2_ emissions. We found that positive and negative shocks to GDP reduce CO_2_ emissions by −0.739 and −1.449 in the long run. This means that a 1 % increase/decrease in GDP should decrease CO_2_ emissions by 0.73 % and 1.44 % in the long run. Several studies have mentioned the impact of economic growth on carbon dioxide emissions. In this article, economic development plays a role in mitigating carbon emissions. Greenhouse gas (GHG) emissions in the Republic of Congo will follow cyclical trends. However, better governance will significantly reduce polluting emissions in the Republic of Congo. Therefore, strengthening governance programs and improving institutional quality generally contribute to reducing greenhouse gas (GHG) emission levels in the Republic of Congo. Then, the Congolese government must promote bioenergy innovation and improve economic growth by developing more renewable energy sources that reduce the ecological footprint. This conclusion is consistent with the results of refs [62,67,69,102].

Similarly, as shown in Table 8, the estimation of the BEC coefficients decomposed of positive and negative shocks in the long run are −1.145 and 0.086, respectively. The finding indicates that negative shock and positive shock to the BEC are significant at the 1 % level, which means that a 1 % increase in BEC should decrease CO_2_ by 1.14 %, while a 1 % reduction in BEC should increase CO_2_ emissions by 0.08 %. The biomass energy consumption in the Republic of Congo does not contribute enough to CO2 emissions; it could be a better solution to achieve a sustainable climate. In this case, the negative shock to BEC forces policymakers and the government to promote the production of renewable energies while valuing the production laws, which include reducing the pollution tax, an economic issue that would lead to an increase in CO_2_ emissions. Therefore, the Congolese government should also pursue policies that should encourage the maintenance of environmental stability. Or achieve energy production and ecological balance. These results are consistent with the findings of [78,82,90,100,108,118,119].

We found that positive shocks to RL and Ge positively impact CO_2_ emissions in the Republic of Congo. While the negative shocks to RL and GE have a negative impact on the country. For a 1 % increase in RL and GE, CO_2_ increased by 0.09 % and 0.10 %, respectively. For a 1 % decrease in RL and GE, CO_2_ decreased by 0.036 % and 0.48 % respectively. This implies that economic reforms have considerably affected the quality of the environment. This is in line with the results of [131,132]. As we can see, in the short term (still in Table 7), the factors are broken down for the positive and negative shocks. Regarding GDP and BEC, we found that a 1 % decrease in each increased the CO_2_ by 0.374 and 0.656 in the short-run, respectively, while a 1 % increase in GE increased CO_2_ by 1.102 in the short-run. Our results are consistent with the authors' study [86,133,134]; they investigated the relationship between economic development, renewable energy consumption, and carbon emissions. For them, three factors of economic growth affect the environment. The first is the scale effect. They show that increased economic activity without energy innovation is in cahoots with increased demand for natural resources, leading to increased pollution and CO_2_ emissions. In this case, economic prosperity positively contributes to the environment. Then is the composition effect. This means that a transformation in the energy manufacturing system's structure accompanies a country's development. In the industrialised nations, environmental deterioration has increased with the structural shift of the economy from rural to urban areas; however, it has been reversed with the structural change from energy-intensive and low-carbon companies to service technologies based on knowledge of energy and climate impacts. The last is the technical effect. They show that when the country embarks on research and development (R&D), new technologies replace obsolete ones and ensure good environmental governance.

#### 4.1.6 Stability diagnostic

**Fig. 5:**
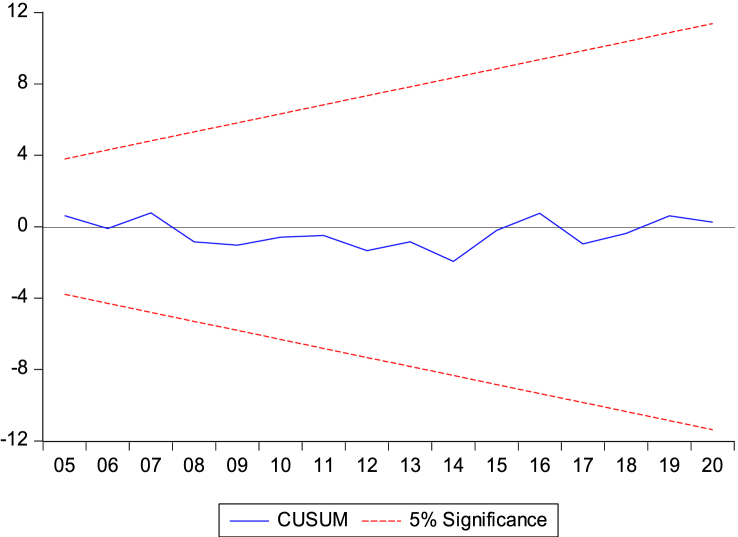
Stability and reliability of the model using Cumulative sum test.

**Fig. 6:**
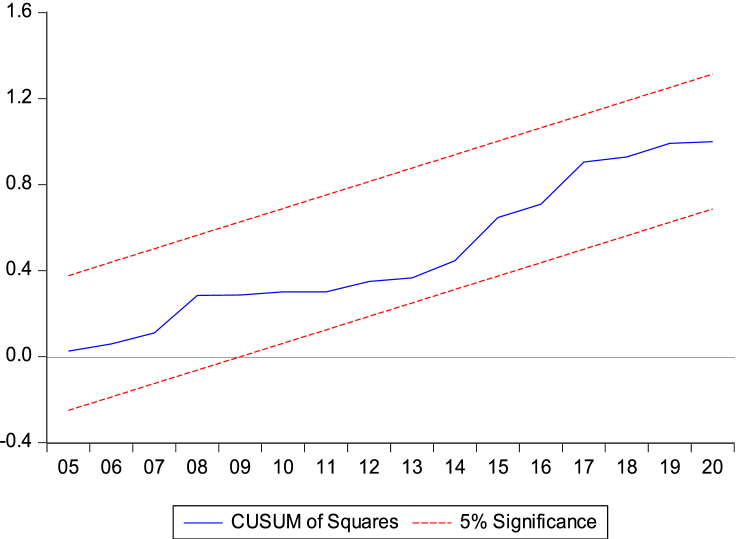
Stability and reliability of the model using Cumulative sum of the squares

We performed the Cumulative sum test and the Cumulative sum of the squares to assess the stability of the model. Therefore, Fig. 5, Fig. 6 below show the stability and reliability of the model. The results show that the evolution of our variables (the blue interval) does not exceed the confidence interval (red interval). The figures show that the estimated model is within the 5 % significance level, indicating that our model is correctly specified and stable in the Republic of Congo.

#### 4.1.7 Graphical presentation of dynamic multiplier diagrams

**Fig. 7:**
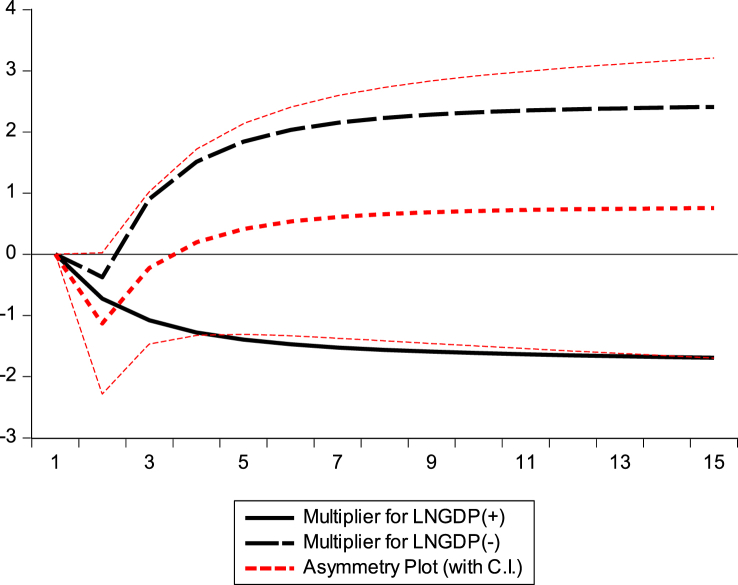
Dynamic multiplier charts of GDP

**Fig. 8:**
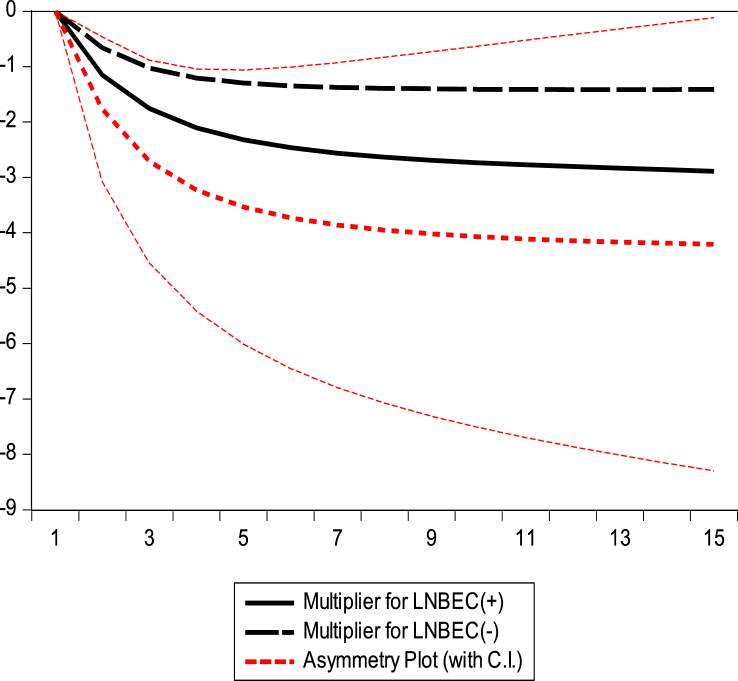
Dynamic multiplier charts of BEC

**Fig. 9:**
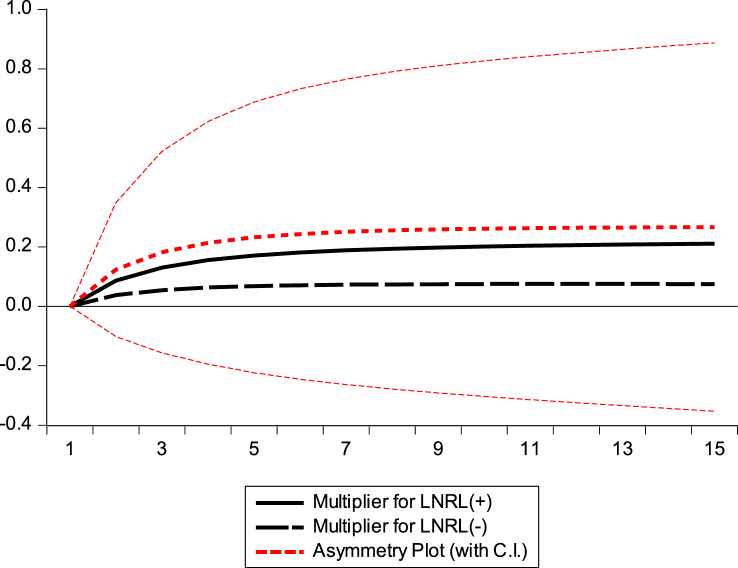
Dynamic multiplier charts of RL

**Fig. 10:**
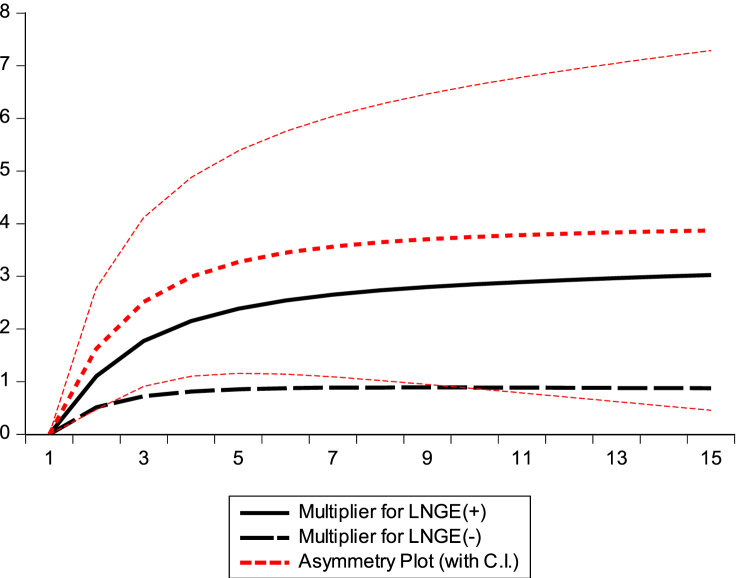
Dynamic multiplier charts of GE

This section examines the asymmetric impact trajectories of GDP, BEC, NR, and GE on climate change in the Republic of Congo. The black solid lines of the dynamic multiplier charts GDP, BEC, RL, and GE in Fig. 7, Fig. 8, Fig. 9, Fig. 10 represent the positive shocks of GDP, BEC, RL, and GE. The black dotted lines represent the negative GDP, BEC, RL, and GE shocks. The red dotted line shows the dynamic multiplier combination due to positive and negative GDP, BEC, RL, and GE shocks on CO_2_ emissions. The vertical axis represents the range of positive and negative shocks, and the horizontal axis represents the period. The results of the dynamic cumulative multiplier plots in Fig. 7, Fig. 8, Fig. 9, Fig. 10 show that the impact of GDP, BEC, RL, and GE on CO_2_ have significant non-linear relationships with climate change in the Republic of Congo.

This confirms the stability of the cumulative sum/cumulative square test model Fig. 5, Fig. 6, the Wald test in Table 6, and the results of the NARDL model in Table 8.

### 4.2 Discussion

The Republic of Congo's unique context distinguishes this study from studies conducted in other countries or regions facing similar environmental challenges. This study focuses on a developing African country with distinct geographical, economic, political, and ecological characteristics. As a result, the socioeconomic and environmental factors influencing climate sustainability may vary from those observed in other parts of the world. Using a NARDL approach, this study investigates the roles of economic growth, biomass energy consumption, rule of law, and government effectiveness in this specific context, providing a thorough and contextual analysis of climate dynamics in this region. Furthermore, this study seeks to demonstrate the superiority of its methodological approach and findings compared to existing literature. Using the NARDL model, this study takes an innovative approach to exploring non-linear relationships between economic, political, and environmental variables, resulting in a better understanding of the underlying mechanisms of climate sustainability. The findings of this study are supported by rigorous empirical analyses, such as Wald test verification and stability diagnostics, which improve the validity and reliability of the conclusions reached.

The results of our NARDL model demonstrated a long-run relationship between economic growth, biomass energy consumption, Rule of Law, governance effectiveness, and climate change. Verifying the Wald test reveals a long-term relationship between the variables in the model. Stability diagnostic within the 5 % significance level and dynamic multiplier diagrams confirmed the results of the NARDL model in Table 8. It can be inferred that economic growth, biomass energy consumption, economic growth rule of law, and government effectiveness have significant non-linear relationships with climate change in the Republic of Congo. The results showed that a 1 % increase/decrease in GDP should decrease CO_2_ emissions by 0.73 % and 1.44 % in the long run. Therefore, strengthening governance programs and improving institutional quality generally contribute to reducing greenhouse gas (GHG) emission levels in the Republic of Congo. This is consistent with the authors [62,67,69,102].

The results also show that a 1 % increase in BEC should decrease CO_2_ by 1.14 %, while a 1 % reduction in BEC should increase CO_2_ emissions by 0.08 %. Indeed, biomass has undeniable assets in the fight against the Congolese greenhouse effect. It is renewable as an energy source since plants absorb the CO_2_ emitted during combustion for their growth. It could be transformed into energy (heat, electricity, fuel) and considered a raw material for materials such as wood in construction, for example, and chemistry [36,38]. Its adaptation to the Republic of Congo also makes it possible to reduce its energy dependence on fossil fuels because of their low energy content, replace petrochemical products with bioproducts that are less harmful to health and the environment, and maintain and develop employment, particularly in rural areas. In addition, an increase in the biomass energy consumption variable would increase economic growth.

In addition, the effect on CO_2_ emissions varies depending on whether there are positive or negative shocks on the variables. The results show that the positive shocks to RL and GE positively impact CO_2_ emissions in the Republic of Congo. While the negative shocks to RL and GE have a negative impact on the country. For a 1 % increase in RL and GE, CO_2_ increased by 0.09 % and 0.10 %, respectively. For a 1 % decrease in RL and GE, CO_2_ decreased by 0.036 % and 0.48 % respectively. This implies that economic reforms have considerably affected the quality of the environment, which is in line with the results of [131,132]. Under the Kyoto Protocol, the Republic of Congo has committed to limiting its greenhouse gas emissions to 5 %. Our results are consistent with previous research showing that economic growth, biomass energy use, rule of law, and government effectiveness influence climate change [52,67,73,74,77,95,96,103,111]. Increases in greenhouse gas emissions often accompany economic growth. This results in an increase in energy consumption, which usually comes from fossil fuels. The results show that increasing biomass energy consumption can help reduce carbon dioxide emissions. This result is consistent with previous research showing that biomass can be an efficient and sustainable energy source. Biomass is a renewable energy source that helps reduce greenhouse gas emissions. Burning biomass releases carbon dioxide into the atmosphere, but the plants absorb it as they grow. Therefore, burning biomass does not lead to increased carbon dioxide levels in the atmosphere. However, it is essential to note that biomass energy consumption can also negatively impact the environment, such as deforestation and land degradation. Therefore, it is necessary to take steps to minimise these effects.

The findings also suggest that the rule of law and government effectiveness can significantly impact climate change. Improving the rule of law and government efficiency can help reduce greenhouse gas emissions by promoting effective environmental policies [125,129]. However, it is essential to note that the impact of the rule of law and government effectiveness on climate change may vary depending on the specific context. For example, in countries with weak rules of law, improving government efficiency may not be enough to reduce greenhouse gas emissions. Suppose a weak rule of law and effective government are to help reduce greenhouse gas emissions. In that case, they must promote effective environmental policy implementation, strengthen environmental institutions' capacity, and increase transparency and accountability.

Research on the role of economic growth, biomass energy use, rule of law, and government effectiveness in climate change remains relatively limited. There are several areas where information or data is lacking. Most studies on this topic have been conducted on relatively small samples. It would be interesting to conduct a study with a larger sample to confirm the results obtained. Most research on this topic has been conducted in developed countries. It would be interesting to conduct research in developing and underdeveloped countries with different economic and environmental conditions. Again, most studies on this topic use traditional statistical methods. Using more advanced techniques, such as network analysis, would be interesting to understand the relationships between different variables better. Finally, most studies on this topic examine a small number of variables. It would be interesting to investigate other variables, such as demographics, technology, and environmental policy, to understand climate change's effects better. The Republic of Congo is expected to consider these findings and should do everything possible to achieve its climate change reduction targets. Finally, this study contributes significantly to climate sustainability research by providing context-specific insights into the Republic of Congo. The study's recommendations and conclusions can help policymakers and practitioners develop policies and strategies to promote climate sustainability in the region and contribute to the scientific literature on this crucial topic.

## 5 Conclusion, policy recommendations, policy implications, and limitations

### 5.1 Conclusion

The Republic of Congo is a country that faces several challenges, including energy, economic growth, the environment, government efficiency, and climate stability. Energy is a significant challenge for Congo, as the country relies heavily on fossil fuels for power generation, transportation, heating, and construction. This reliance on fossil fuels is costly and contributes to climate change. Economic growth is another major challenge facing Congo. The country needs economic growth to create jobs and reduce poverty. However, economic growth could also increase energy consumption and greenhouse gas emissions. Government effectiveness is a significant challenge in Congo due to neglectful decision-making and weak governance. The inefficiency of the government makes it difficult to implement effective public policies to address the other challenges facing the country. Climate stabilisation is a significant challenge for Congo, as the country is particularly vulnerable to the effects of climate change. Climate change can lead to droughts, floods, storms, and other extreme weather events, negatively impacting Congo's economy, environment, and people. These challenges are interrelated. Our research focused on the role of economic growth, biomass energy consumption, rule of law, and government effectiveness in achieving climate sustainability in the Republic of Congo.

The NARDL model results show a long-term relationship between economic growth, biomass energy consumption, the rule of law, governance effectiveness, and climate change in the Republic of Congo. The Wald test confirms this relationship, and stability diagnostics and dynamic multiplier diagrams support the findings. Economic growth, biomass energy consumption, rule of law, and government effectiveness have all demonstrated significant nonlinear relationships with climate change. In particular, a 1 % increase/decrease in GDP should reduce CO2 emissions by 0.73 % and 1.44 % in the long run, demonstrating that improving governance programs and institutional quality can reduce greenhouse gas emissions. Furthermore, rising biomass energy consumption is linked to lower CO2 emissions, as biomass is a renewable energy source that absorbs emitted CO2 during plant growth. Furthermore, positive shocks to the rule of law and government effectiveness raise CO2 emissions, highlighting the importance of effective environmental policies and governance reforms. These findings are consistent with previous research demonstrating the impact of economic growth, biomass energy use, the rule of law, and government effectiveness on climate change. Our findings also show that increasing BEC in the long run can reduce CO2 emissions and may represent a viable alternative to fossil fuels. Increased focus on BEC research and development might result in more efficient and sustainable biomass conversion technologies, increasing their climate benefits. Promoting BEC has the potential to stimulate local economies by creating jobs in the forestry, agriculture, and biofuel production sectors. This is consistent with our goals of climate sustainability and economic growth. Promoting biomass consumption has the potential to significantly boost local economies by creating jobs in key sectors like forestry, agriculture, and biofuel production. This approach not only diversifies energy sources and reduces reliance on fossil fuels but also encourages the development of biomass conversion technologies, stimulating innovation and investment in the region. Furthermore, promoting biomass use helps to increase ecosystem resilience by encouraging sustainable agricultural and forestry practices and creating job opportunities in these sectors. This approach, which integrates climate sustainability goals with local economic growth objectives, effectively achieves sustainable and equitable development. Valorising biomass provides the Republic of Congo with a path to increased energy security by reducing its reliance on imported fossil fuels. By prioritising biomass as an energy source, the country can reduce the risks associated with oil price volatility and global market fluctuations. Furthermore, the Republic of Congo can stabilise its energy supply and ensure a consistent fuel supply to meet its internal needs. The strategy thus helps to strengthen the country's energy independence, reduce vulnerability to external shocks, and promote more sustainable and self-sufficient management of its energy resources. Furthermore, by investing in local biomass production, the Republic of Congo can boost economic growth by creating jobs in forestry, agriculture, and biomass processing while improving its ability to address global energy challenges.

To address these challenges, the Congolese government must adopt a holistic approach considering the interdependence between these different aspects. Public policies should promote sustainable economic growth, improve government efficiency, and mitigate the effects of climate change. The Republic of Congo must encourage the emergence of biomass energy consumption to promote its economic growth and climate stability. This energy consumption in the Republic of Congo is expected to remain very high and grow in the coming years because the Republic of Congo has vast potential sources of biomass: Congo Basin forests, agricultural residues, wastewater, industrial residues, animal residues, and municipal solid waste, to name a few.

### 5.2 Policy recommendations

In addition, given the results of our study, the recommendations are as follows: Given that our results show the asymmetric effects of economic growth, biomass energy consumption, rule of law, and government effeteness on CO_2_, Congolese policymakers should take into account the changing effect of the included explanatory variables on CO2 emissions. This requires the Congolese government to continuously monitor the impact of selected variables and take action as early as possible.

The results suggest that economic growth can improve the quality of the environment or climate in the Republic of Congo. However, we see that daily life in the Republic of Congo is still affected by fossil fuels. However, given the positive and negative shocks of economic growth on CO_2_ emission, Congolese policymakers should modify energy consumption, improve energy efficiency, and then replace the consumption of fossil fuels with renewable energies that are less polluting and allow decentralisation of energy production. Bringing fossil fuels into the Congolese energy mix, the government faces many challenges that need to be met over time: investments and substituting certain existing assets for a holy economic development in CO_2_ emission. Review the development policy formulation strategy annually to avoid fraudulent practices that slow economic growth, seek and ensure political stability, and implement effective long-term economic policies.

Congolese policymakers must consider that the effects of biomass energy consumption on CO_2_ can be assessed as the most suitable variable for reducing carbon emissions in the Republic of Congo. Biomass is the oldest source of energy mastered by man and is today the first source of renewable energy capable of meeting the climate challenge. Therefore, the biomass energy consumption variable must be considered in the policy development and implementation.

Given the asymmetric effects of biomass energy consumption on CO_2_, Congolese policymakers should focus on positive shocks and not negative shocks. Positive shock has a negative impact, meaning that when biomass energy consumption increases automatically, CO2 emission decreases. Therefore, it is essential to ensure and maintain biomass energy consumption by avoiding any problems affecting its supply.

Congolese policymakers should increase the rule of law and government effectiveness to limit CO_2_ emissions. The current level does not fully allow the Republic of Congo to exercise environmental improvement. Their consideration must be known in ecological planning. So, policymakers should put more effort into achieving the rule of law and government effeteness stability to support innovation and energy technologies to banish the negative effects of the rule of law and government effeteness on climate.

Congolese policymakers should focus on all factors that influence CO_2_ emissions at the same time. Not focusing on just one aspect and a harmonious approach to managing influential factors is meaningful. With the diversity of economic growth, biomass energy consumption, the rule of law, and government effeteness on CO2 reduction, Congo must adapt to the current energy context and favour partnerships with the actors concerned: resource producers, technology suppliers, industrialists, and academic research.

### 5.3 Policy implications

The policy implications of our research are diverse.

At the national level, your research shows that the Congolese government is essential in reducing greenhouse gas emissions and combating climate change. In particular, governments must Implement environmental policies such as fossil fuel taxes, emissions standards, and energy efficiency programs. They are improving the rule of law and government efficiency, strengthening environmental institutions, increasing transparency and accountability, and fighting corruption. These measures require strong political commitment from the Congolese government. In addition, various stakeholders, including business, civil society, and international organisations, should be consulted.

At the international level, your research shows that Congo can be essential in combating climate change. Congo is a country rich in natural resources, especially biomass resources. Developing biomass as a renewable energy source can help reduce global greenhouse gas emissions. Congo can also contribute to the fight against climate change by participating in international climate negotiations. The country could commit to ambitious measures to reduce greenhouse gas emissions and provide financial support to developing countries to implement these measures.

At a local level, your research can help raise awareness of climate change issues among Congolese people. They can also help mobilize support for government action on climate change. This research could also be used to support the development of pilot projects for biomass development and strengthen the rule of law. These pilot projects can be examples of developing more ambitious policies and programs.

In the long term, this research could positively impact Congo's climate stability. It helps strengthen the political commitment of the Congolese government, promotes international cooperation, and raises public awareness of the challenges of climate change.

### 5.4 Limitation and future study

This study focuses only on the Republic of Congo, with a significant trend in study variables due to data availability constraints. Comparative analysis of Congo with other countries would be an excellent approach for future research. In our study, we touched on several types of biomasses. However, in the future, researchers can focus on every kind of biomass separately from the others to see which has a more positive or negative effect on economic growth and the environment. Finally, it would also be interesting for future researchers to research the link between Foreign Direct Investment and their interaction with energy consumption, economic growth, and variables associated with the sustainable development of CO_2_ emissions based on the controversial hypothesis of Grossman and Krueger. Future research may also consider the impact of economic growth on climate change in developing countries. The effect of biomass energy consumption on climate change in a context of weak rule of law. The impact of the rule of law and government effectiveness on climate change in countries with different economic and environmental conditions. You can also use more advanced statistical methods like network analysis to better understand the relationships between various variables. Further research into the relationship between ecological sustainability and biological mechanisms, such as the role of small non-coding RNAs (piRNAs), can advance our understanding of complex ecosystem dynamics and inform long-term climate resilience strategies.

## Funding

This research was funded by the 10.13039/501100001809National Natural Science Foundation of China (51874003).

## Data availability Statement

The data used and their sources are provided in the paper.

## CRediT authorship contribution statement

**Railh Gugus Tresor Massonini Ngoma:** Writing – review & editing, Writing – original draft, Methodology, Investigation, Formal analysis, Data curation, Conceptualization. **Xiangqian Wang:** Visualization, Supervision, Project administration, Methodology, Funding acquisition, Conceptualization. **Xiang Rui Meng:** Visualization, Validation, Project administration, Conceptualization. **Cety Gessica Abraham Mahanga Tsoni:** Writing – review & editing, Writing – original draft, Methodology, Investigation, Data curation. **Sumaiya Bashiru Danwana:** Writing – review & editing, Writing – original draft, Methodology, Formal analysis. **Benjamine Tsoni Ndombi:** Data curation, Methodology, Writing – review & editing.

## Declaration of competing interest

The authors declare that they have no known competing financial interests or personal relationships that could have appeared to influence the work reported in this paper.
